# The Effects of the Environment on the Drawings of an Extraordinarily Productive Orangutan (*Pongo pygmaeus*) Artist

**DOI:** 10.3389/fpsyg.2019.02050

**Published:** 2019-09-06

**Authors:** Yuki Hanazuka, Hidetoshi Kurotori, Mika Shimizu, Akira Midorikawa

**Affiliations:** ^1^Institute of Cultural Sciences, Chuo University, Tokyo, Japan; ^2^Tama Zoological Park, Tokyo, Japan; ^3^Department of Psychology, Faculty of Letters, Chuo University, Tokyo, Japan

**Keywords:** drawing behavior, evaluation of drawings, environmental effects, orangutan (*Pongo pygmaeus*), aging

## Abstract

We report on the case of an extraordinary orangutan who spontaneously produced over a thousand drawings in 5 years. This female orangutan, Molly, started drawing when she was estimated to be 50 years old. Although it has been established that great apes spontaneously draw without training, she produced an enormous number of paintings in her old age, and the numbers of lines and colors in her drawings varied from day to day. As her drawings seemed to be affected by her surroundings, we attempted to analyze quantitatively relationships between her drawings and potentially influential factors during a specific period in which no ostensibly major events were observed. According to our results, her drawings were affected by the identity of her keeper, implying that her drawing behavior may have been affected by environmental factors. Thus, drawings may serve as windows to the internal states of non-human primates.

## Introduction

Drawing is a behavior observed not only in humans but also in enculturated great apes (Morris, 1962), capuchin monkeys (Morris, 1962), and an Asian elephant (Gucwa and Ehmann, 1985). Although the elephant produced a drawing when it was rewarded, captive great apes are known to spontaneously produce “reward-free” drawings without special training or rewards (Schiller, 1951; Morris, 1962; Boysen et al., 1987). Therefore, drawing could be a form of enjoyable activity or self-gratifying play in great apes (Lenain, 1995). However, to our knowledge, non-human primates have never produced representational drawings. Indeed, previous studies have examined drawing behavior as a means to investigate perceptual organization in primates (Schiller, 1951; Saito et al., 2014), implying that such drawing behavior reflects only one mode of the cognitive function of non-human primates. Additionally, research on human children found that they select the colors used in their drawings based on their emotional state when they are drawing (Crawford et al., 2012). Based on the foregoing, it is plausible that drawings reflect the internal state not only of human but also non-human primates. However, it has been difficult to analyze relationships between the drawings and the internal states of non-human primates because of the limited number of drawings produced by non-human primates, and opportunities to observe associations between the content of their drawings and their daily lives are rare. Fortunately, we had the opportunity to explore associations between the drawings and the daily life events of an orangutan, Molly, who produced an extraordinary number of drawings (over 1,037). By quantitatively analyzing her drawings using the semantic differential (SD) method and multiple regression analysis, we confirmed three episodes in which the content of her drawings seem to have changed as a function of daily events.

## Case Report

Molly was a Bornean orangutan (*Pongo pygmaeus*) who was probably born in the wild in 1952. Until 1955, she was reared in Ueno Zoological Gardens, Tokyo, Japan, and she was moved to the Tama Zoological Park in 2005 (when she was approximately 53 years old). Molly was housed separately and rarely had direct contact with conspecifics. However, she regularly had the opportunity to see other individuals.

In 2002, when she was 50 years old and still housed at Ueno Zoological Gardens (Tokyo Zoological Park Society, 2002), Molly began to draw at random times. In 2005, at age 53 years, Molly was moved to the Tama Zoological Park. Beginning in July 2006, Molly had the opportunity to draw irregularly, and then in April 2007 she began to draw routinely as part of a behavioral enrichment program started by her keepers because she spent so much time alone; this program did not include food or water deprivation. Molly was housed in an outdoor or indoor enclosure in the morning, and then produced drawings after returning to the resting room in the afternoon.

**FIGURE 1:**
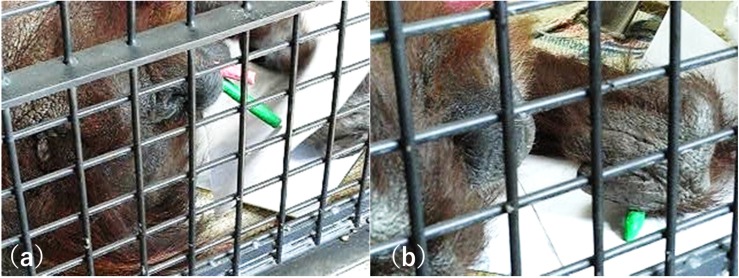
**(a)** Molly is holding red and green crayons in her mouth and is preparing to draw. **(b)** Molly is also holding a green crayon in her left hand and is drawing on the paper.

The keeper provided Molly with a piece of high-quality paperboard and variously colored crayons in buckets, and she was allowed to draw freely at any time (Figure 1). On average, Molly spent 2 or 3 h drawing and produced one or two drawings each day. When Molly finished her drawings, she placed the drawing supplies on the floor. Molly created over 1,037 drawings over a period of 5 years. The enrichment program was non-invasive and was carried out in a resting room (1.8 m wide × 2.6 m deep × 2 m high) surrounded by a mesh fence (each hole in the mesh measured 5 × 5 cm). When Molly was drawing, the keeper stood nearby or cleaned another resting room near Molly’s room.

**FIGURE 2:**
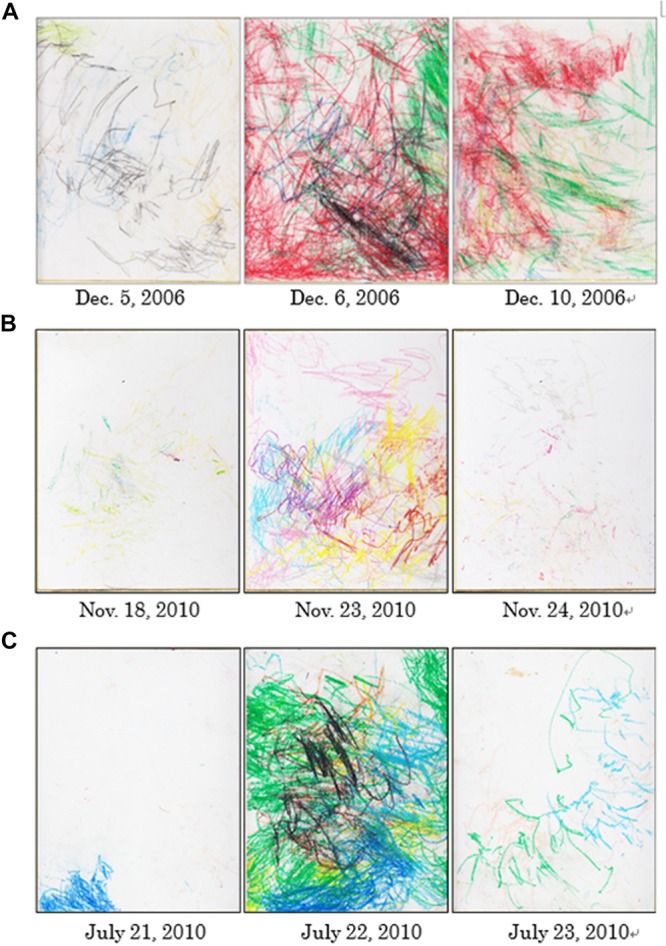
**(A)** The drawings produced by Molly the day before the delivery, the day of the delivery, and the day after the delivery. **(B)** The drawings produced by Molly the day before received a new set of crayons, the day she received them, and the day after she received them; and **(C)** the drawings produced by Molly the day before the visit by elementary school students, the day of the visit by elementary school students, and the day after the visit by elementary school students.

The drawings produced by Molly seemed to reflect daily events in her life. For instance, on December 6, 2006, an unrelated female orangutan gave birth. Molly was resting in her room during the birth, which allowed her to figure out what was happening even though she could not directly view the birth. Figure 2A presents the drawings Molly produced on the days before, of, and after the delivery. The drawings produced on the days of the birth and after the birth contained copious amounts of red, suggesting that her drawing behavior may have been influenced by the smell of blood and the excited state of other individuals at the time of delivery. On November 23, 2010, Molly received a new set of crayons for drawing. Figure 2B depicts the drawings she produced the day before she received the new crayons, on the day she received them, and on the day after she received them. The numbers of lines and colors were especially varied on the day she received the crayons. It is possible that the introduction of a novel drawing tool may have increased her motivation to draw. Furthermore, on July 22, 2010, elementary school students visited the zoo to learn about the job of an animal keeper. Figure 2C presents the drawings produced by Molly on the day before their visit, the day of their visit, and the day after their visit. These drawings also reflect an especially variegated use of lines and colors. Thus, Molly may have been activated by the unusual presence of elementary school students.

These episodes imply that Molly’s drawing behavior was affected by daily events. We used the semantic differential (SD) method to evaluate quantitatively the content of her drawings and to investigate the kinds of environmental changes that were related to such content. We then performed a multiple regression analysis to identify more precisely the factors that affected the content. Moreover, the drawings used for analysis were not selected based on whether they were produced during the occurrence of a particular environmental change or event; instead, they were pseudo-randomly selected from drawings produced during Molly’s ordinary daily life.

## Methods and Results

### Study 1: Evaluation of Orangutan Drawings

Study 1 evaluated the orangutan drawings quantitatively using SD methods based on subjective evaluations by 61 humans (see the Supplementary Material for the detailed methods).

**FIGURE 3:**
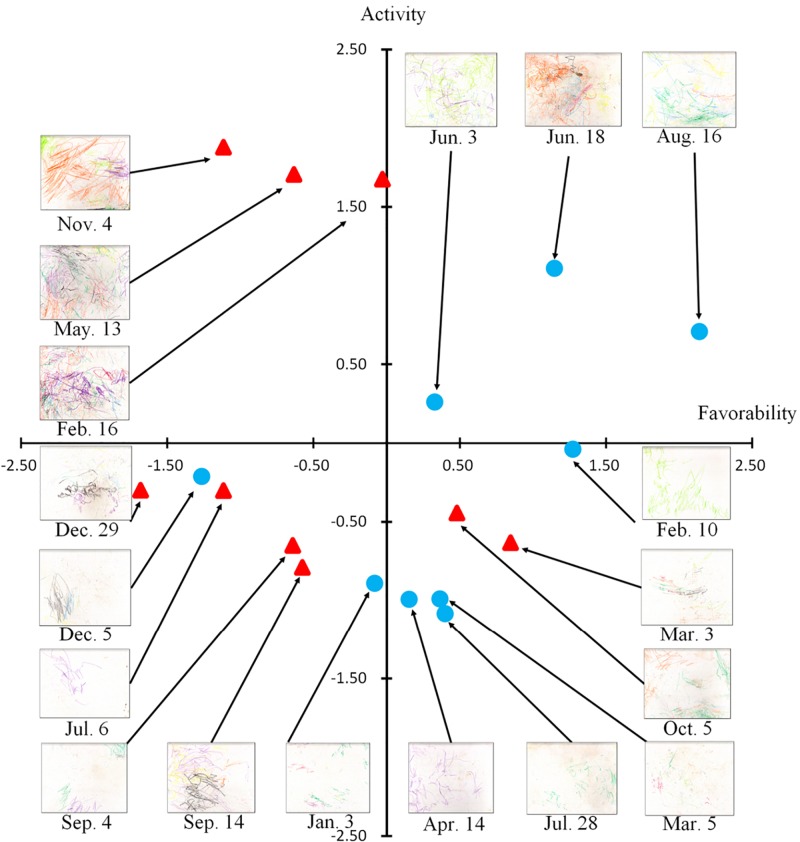
Results of the factor analysis. The horizontal axis reflects the favorableness score, and the vertical axis reflects the activity score. The drawings by the orangutan are located relative to the two dimensions. The date under each drawing indicates the day it was produced, between April 2006 and March 2007. The blue circle indicates a day Molly produced the drawings in the presence of the older male keeper. The red triangle indicates a day Molly produced the drawings in the presence of the younger female keeper.

Two factors were determined to be statistically significant (Figure 3 and Supplementary Appendix 4). Factor 1 (45.53%) was named activity (excited–calm, lively–sober, dynamic–static, and blunt–sharp), consisting of a variety of colors and sharp lines. Factor 2 (44.59%) was named favorability (pleasant–unpleasant, likable–repugnant, beautiful–ugly, soft–hard, and smooth–rough), consisting of warm colors and curved lines. Repeatability analysis (Cronbach’s alpha) showed a sufficient level of reliability (activity: 0.95, favorability: 0.93). Supplementary Appendix 2 shows the scores of the drawings resulting from factor analysis.

The activity factor was characterized by a variety of colors and sharp lines, indicating that the orangutan had repeatedly changed crayons and moved her hands up and down. Favorability was associated with warm colors and curved lines, which are thought to be more complex than straight lines (Tanaka, 2003).

### Study 2: Influence of Keeper Presence on Orangutan Drawings

Study 1 demonstrated that the orangutan drawings were composed of two evaluative factors. Based on these results, we attempted to determine whether these factors were affected by the presence of either of two familiar keepers using the same 18 drawings as in Study 1 (see the Supplementary Material for the detailed methods).

The results of our multiple regression analysis showed that none of our candidate predictors was related to activity scores [*F*_(__3__,__17__)_ = 0.87, *n. s.*]. Conversely, the keeper was a significant predictor of the favorability score [*F*_(__3__,__17__)_ = 3.51, *p* < 0.05; Supplementary Appendix 3], implying that the presence of male keeper improved the evaluator reactions to the drawings. No other predictors were found to be significant for favorability scores. As a result, we were able to determine whether the identity of Molly’s keeper affected the scores accorded to the drawings.

## Discussion

Molly began drawing when she reached an advanced age, and she produced over 1,000 drawings in the 5 years before she died. Her drawings seemed to express daily changes in her environment. Moreover, the SD and multiple regression analyses revealed that her drawings changed according to the identity of her keeper. These results imply that her drawings were not merely *ad hoc* representations of circumstantial changes but might instead reflect the effects of such changes on Molly’s internal state. These findings imply that, similar to humans, non-human primates may draw to express the impact of external changes on internal states.

Why did the identity of the keeper influence Molly’s drawing behavior? Previous studies of human children and chimpanzees have reported that drawing behavior is promoted by the presence of a familiar person (Yamagata, 1988; Tanaka, 2003). In this case, an elderly male keeper had reared Molly for a period of 22 years in the two zoos. Moreover, when Molly met the keeper after 13 years at Tama Zoological Park, she responded to his call and moved close to him. This approach behavior was considered to reflect Molly’s confidence in this keeper, suggesting a trusting relationship between them. In contrast, her other keeper was a young female with a much shorter history with Molly. Smith (2014) found that orangutans initiated more close-affiliative behaviors with familiar humans than with unfamiliar humans, indicating that their behavior changed according to familiarity. Therefore, the presence of a familiar human may explain certain characteristics of Molly’s drawing behavior.

This study has several limitations. First, the sex of the keepers may have affected the drawing behavior of the orangutan. A previous study reported that female Japanese macaques had higher blood pressure and emitted more warning sounds in the presence of human males than in the presence of human females (Koda et al., 1998). It is possible that both familiarity with, and the sex of, the keeper influenced the drawing behavior in this study. Further research on orangutan drawing should control for the sex of the keeper who is present. Second, this study analyzed a small sample of drawings (*n* = 18 of 1037), which might not have reflected all of the characteristics of the drawings produced by the orangutan (Molly) and may limit the generalizability of our findings. Other sample sets should have been prepared to verify that the effect was consistent.

Molly was an extremely rare individual, as she spontaneously produced numerous drawings on a daily basis, creating 1,037 drawings in 5 years. Why did Molly produce so many drawings? Molly was the oldest known orangutan in the world. According to clinical neuroscience research on humans, some demented patients demonstrate painting skills after the onset of the disease (Miller et al., 1998). This suggests that Molly’s prolific output may have been attributable to specific neurodegenerative changes. However, this interpretation does not contradict the possibility that both human and non-human primates express their internal states in their drawings. Indeed, drawing behaviors may offer a means for understanding not only the cognitive abilities but also the internal states of non-human primates.

## Data Availability

The datasets generated for this study are available on request to the corresponding author.

## Ethics Statement

The Tama Zoological Park Ethics Board approved this non-invasive behavioral study, which complied with the Japanese Association of Zoos and Aquariums’ Code of Ethics. This research also complied with the World Association of Zoos and Aquariums Ethical Guidelines for the Conduct of Research on Animals by Zoos and Aquariums.

## Author Contributions

YH and AM developed the study and wrote the manuscript. HK and MS provided the drawing tools to Molly and observed her drawing behavior. YH analyzed the data.

## Conflict of Interest Statement

The authors declare that the research was conducted in the absence of any commercial or financial relationships that could be construed as a potential conflict of interest.
