# Supplementary material for: The Effects of the Environment on the Drawings of an Extraordinarily Productive Orangutan (Pongo pygmaeus) Artist
Source: Front Psychol. 2019 Sep 6;10:2050. doi: 10.3389/fpsyg.2019.02050 (PMC6743438; doi:10.3389/fpsyg.2019.02050)
Supplement: Supplementary file 1 [file Data_Sheet_1.pdf]

SUPPLEMENTARY MATERIALS

1. Study 1: Evaluation of orangutan drawings

1.1. Stimuli

We collected the drawings when Molly set her crayon on the floor. Molly produced a few drawings per month (M =13.83; range: 6–23). Molly produced 112 of the 233 drawings in the presence of keeper A (older male) and the other 121 drawings in the presence of keeper B (younger female) between April 2007 and March 2008. This period was the first year that Molly began to draw routinely as part of a behavioral enrichment program. We randomly selected 9 of the 112 drawings made in the presence of keeper A and 9 of 121 drawings made in the presence of keeper B. Thus, 18 drawings were pseudo-randomly selected as target stimuli. First, we assigned the pseudo-random numbers produced by the Excel pseudo-random number generator to the drawings and sorted them in ascending order. Then, we selected nine drawings in order of the highest to the lowest. The small sample size is a limitation of this analysis; however, we believe a greater evaluation load would have had a significantly negative effect on the outcome because university students were asked to evaluate 18 drawings using each of nine adjective pairs, which required 162 evaluations. Thus, we deemed it necessary to limit our sample. However, based on the findings of a previous study in which 15 adjectives were used to evaluate seven

drawings (Suzuki et al., 2005), we consider that nine drawings were sufficient to meet the minimum necessary requirement for the evaluation and analysis in our study. Moreover, ours was a retrospective study aimed at analyzing a subset of drawings from the collection.

1.2. Procedure

To evaluate the orangutan drawings based on the SD method, we used an evaluation form with nine indices on a seven-point bipolar rating scale (Appendix 1). All indices were based on previous research related to drawings (Suzuki et al, 2005). Three of these indices formed semantic classes, assessed using nine scales: evaluation (beautiful–ugly, pleasant–unpleasant, likable–repugnant), activity (excited–calm, dynamic–static, and lively–sober), and potency (soft–hard, smooth–rough, and blunt–sharp). University students were instructed to use these adjectives to evaluate the orangutan's drawings. The selected adjective had a high loading factor in each semantic class.

We instructed 61 university students aged 18–24 years (M = 19.24, SD = 0.88), 29 males and 32 females, to evaluate 18 drawings on a scale of –3 to +3 using nine pairs of adjectives. The drawings were presented randomly to avoid any systematic effects. The evaluation scores of all participants for each drawing were averaged to obtain a final utility rating. Group evaluation was performed in a lecture room, following a statistics lecture. Participants were informed that the study concerned how people reacted to

drawings made by an orangutan, and their instructions stressed the evaluator's first impressions of the sample drawing. Although, it was necessary to tell the students that the drawings were made by an orangutan, because the present study was conducted as part of a lecture, they were not told whether the keeper was present or which keeper was present when the orangutan produced the drawings. Therefore, the instructions did not alert the participants to the possibility that the presence of a keeper might have influenced the drawing behavior of the orangutan. Moreover, although the assessments were performed during a lecture, the participants could not see others' evaluations because they were not seated next to each other. Therefore, it is unlikely that participants' judgments were influenced by those of others. Participants were then provided with a leaflet containing the instructions and SD scales. The rating range varied from 'strongly agree with the adjective on the left side' to 'strongly agree with the adjective on the right side' (i.e., –3, –2, –1, 0, +1, +2, +3). To avoid bias among the 18 evaluations, we randomized the order of the adjective pairs in the list of semantics. The experimenter presented one of the 18 drawings on a white screen for 30 s, and participants were asked to evaluate each drawing on a senven-point SD scale during the presentation. To avoid fatigue, we provided a 60-s break once half of the drawings had been presented.

1.3. Statistical analysis

To evaluate the orangutan drawings quantitatively, SD data obtained from the experiment were subjected to factorial analysis (FA) using SPSS 11.5 statistical software. FA was conducted on all 18 drawings using the ratings of the nine pairs of bipolar adjectives as independent variables. Principal component analysis with varimax rotation, which has been widely used to analyze impressions of drawings (Suzuki et al., 2005), product designs (Hsu, Chuang, & Chang, 2000), and landscapes (Park, Furuya, Kasetani, Takayama, Kagawa, & Miyazaki, 2011), was conducted as part of the FA. The number of factors was determined considering eigenvalues of the rotated components that exceeded 1.0. The semantic structure was interpreted by including variables with loads exceeding 0.4.

2. Study 2: Influence of keeper presence on orangutan drawings

2.1. Methods

2.2. Predictor variables

The relationships between the two obtained factors and three variables, keeper, location and outside temperature were investigated as follows: 1. Two animal keepers, A and B, cared for the orangutan in 2007 when the drawings were obtained. Keeper A was a 50-year-old man who had cared for Molly for 22 years in total (Ueno Zoological Gardens,

1972–1992; Tama Zoological Park, 2005–2007) and had a longtime relationship with Molly. Keeper B was a 30-year-old woman who had looked after Molly for only 2 years at the Tama Zoological Park (2006–2007), beginning when Molly was approximately 54 years old. Both keepers provided Molly the opportunity to draw under the same conditions. 2. Location data, i.e., whether Molly was housed in an outdoor or indoor enclosure before drawing, was used as a predictor variable because we considered that the outdoor enclosure might provide Molly with more activity options than did the indoor enclosure, which could have influenced Molly's motivation to draw. 3. The average temperature on days when Molly produced drawings was also used as a predictor variable because orangutans in the wild prefer to rest in regions with a consistent temperature (Carne, Semple, & Lehmann, 2012), suggesting that temperature might have influenced Molly's drawing behavior. The average daily temperature for Hino Hachioji, Tokyo, Japan, was obtained from the Japan Meteorological Agency (2014) and was used for analysis.

2.3. Statistical analysis

We performed a multiple linear regression to examine whether environmental conditions predicted the factors produced by the SD method. An average rating score, which we used in study 1 for each drawing was calculated for each factor and used as a dependent

variable in the multiple regression. All analyses were performed using SPSS statistical software. Two-tailed significance was determined at a level of $p<.05$.

	Very	Fairly	Little	Neutral	Little	Fairly	Very	
Beautiful								Ugly
Pleasant								Unpleasant
Likable								Repugnant
Excited								Calm
Dynamic								Static
Gay								Sober
Soft								Hard
Smooth								Rough
Blunt								Sharp

Appendix 1. Example of the questionnaire used to develop a semantic differential measure for each drawing. Participants rated the drawings according to their relationships to nine pairs of contradictory adjectives.

Appendix 2. Factor loadings of nine pairs of adjectives.

Adjectives	Factor 1	Factor 2
Excited－Calm	**0.981**	-0.032
Gay－Sober	**0.978**	0.087
Dynamic－Static	**0.924**	-0.125
Blunt－Sharp	**0.867**	0.322
Likable－Repugnant	0.055	**0.985**
Pleasant－Unpleasant	-0.072	**0.948**
Beautiful－Ugly	0.022	**0.916**
Soft－Hard	-0.481	**0.798**
Smooth－Rough	-0.576	**0.735**
Final statistics		
Eigenvalue	4.098	4.013
Percentage of variance	45.5	44.6
Cumulative percentage	45.5	90.1
Label	Activity	Favorableness

Appendix 3. Multiple regression analysis (forcibly entered) of effects of environmental factors on FA scores.

Variable	*B*	SE *B*	*β*	*t*- *test value*	R^2
Activity					0.16
					$F_{(3, 17)}$=0.87, *n.s.*
Keeper	−0.78	0.57	−0.35	−1.38	
Location	−0.76	0.67	−0.32	−1.13	
Outside temperature	0.02	0.04	0.14	0.51	
Favorability					0.43
					$F_{(3, 17)}$=3.51, $p < 0.05$
Keeper	0.66	0.22	0.62	2.99**	
Location	0.45	0.26	0.40	1.70	
Outside temperature	0.00	0.01	0.01	0.03	

**$p < 0.01$.

Appendix 4. Larger drawings examined with the SD method and multiple regression analysis.

(a) The first quadrant in figure 3

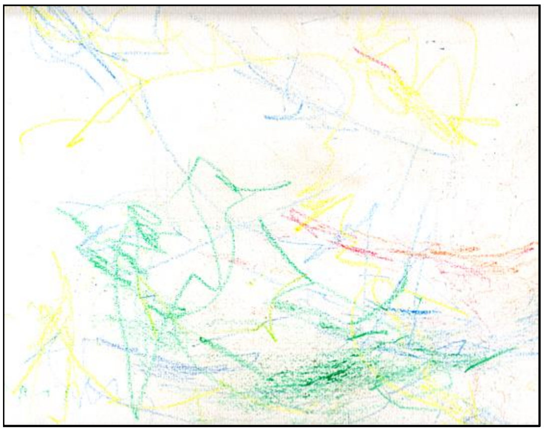

Aug.16

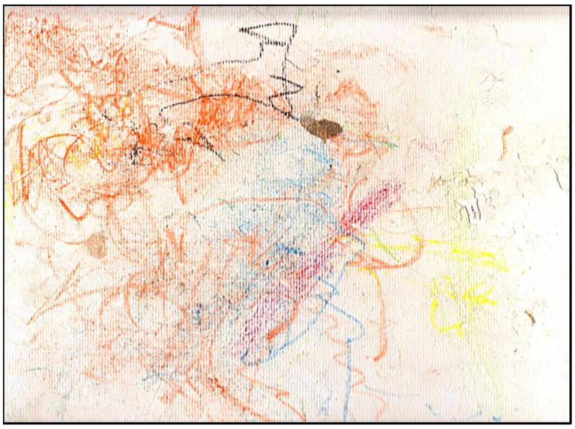

Jun.18

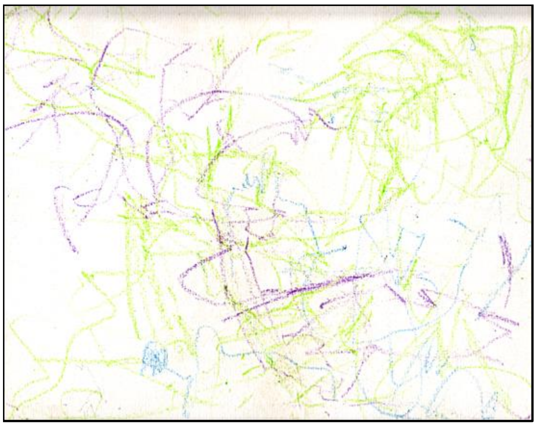

Jun. 3

(b) The second quadrant in figure 3

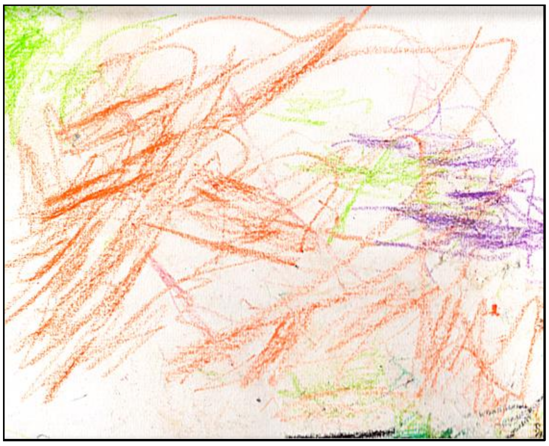

Nov. 4

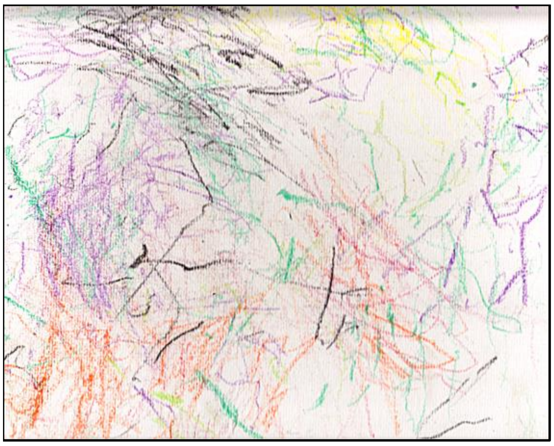

May. 13

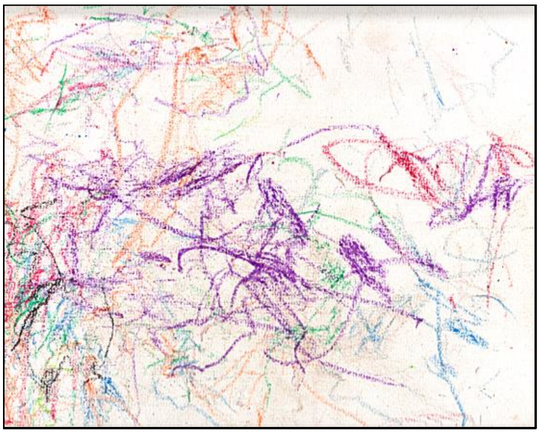

Feb. 16

(c) The third quadrant in figure 3

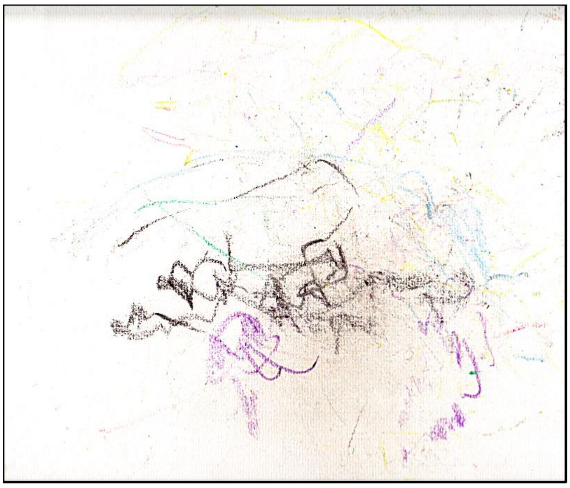

Dec. 29

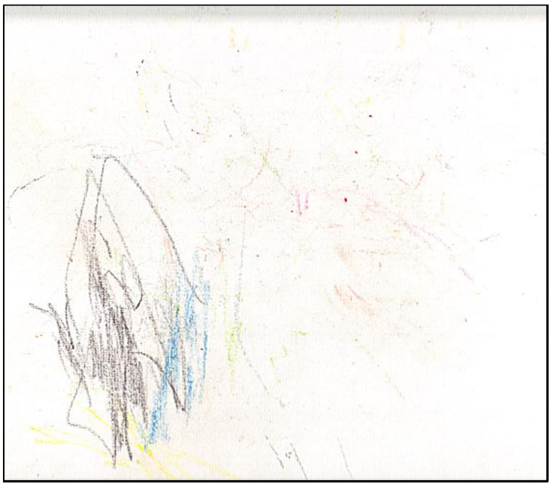

Dec. 5

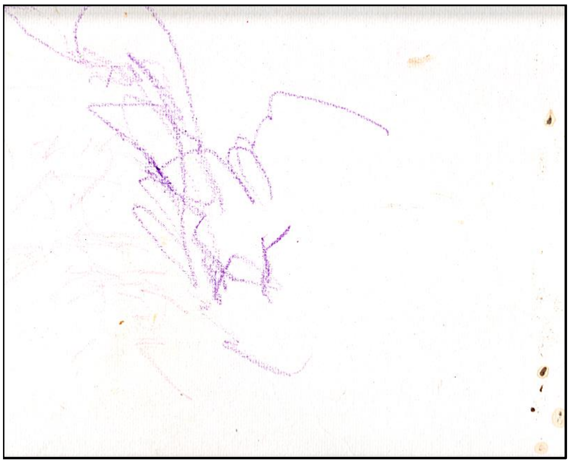

Jul. 6

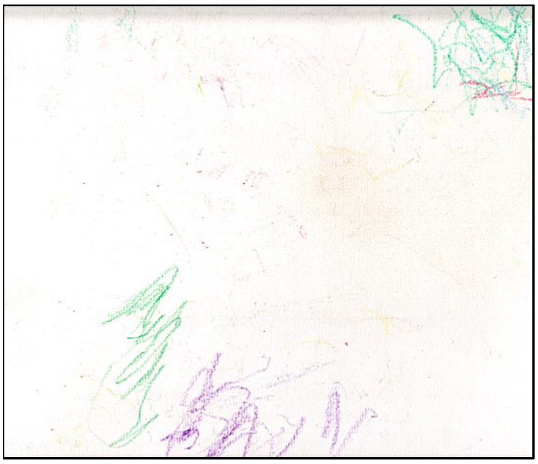

Sep. 4

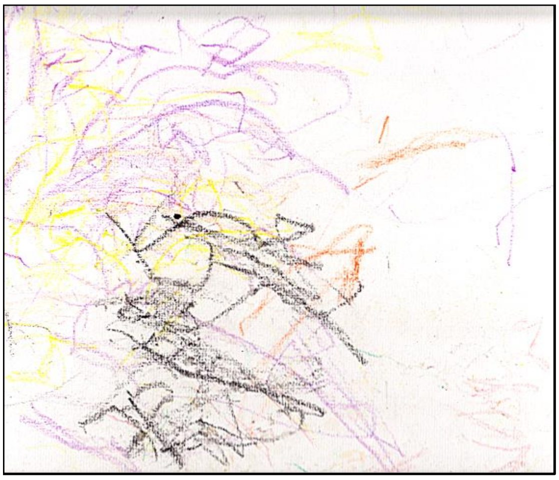

Sep. 14

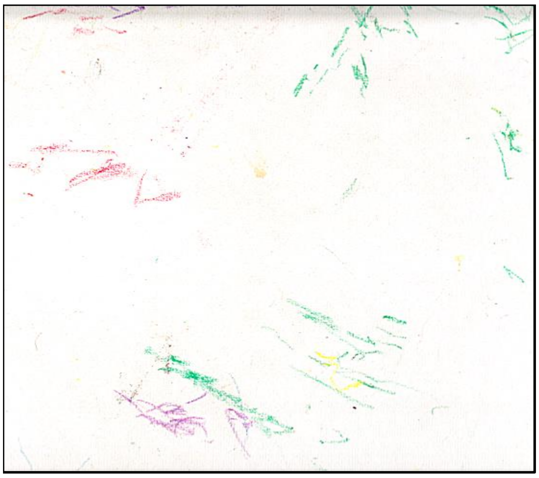

Jan. 3

(d) The fourth quadrant in figure 3

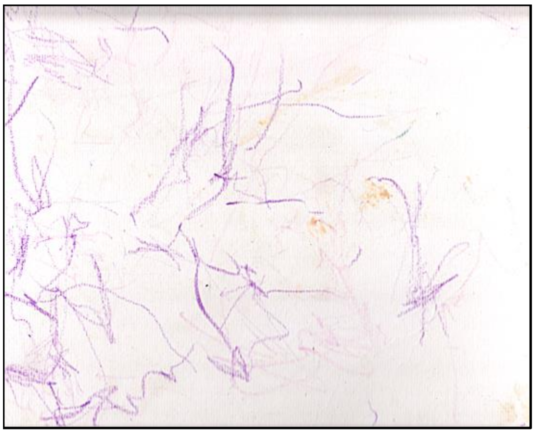

Apr. 14

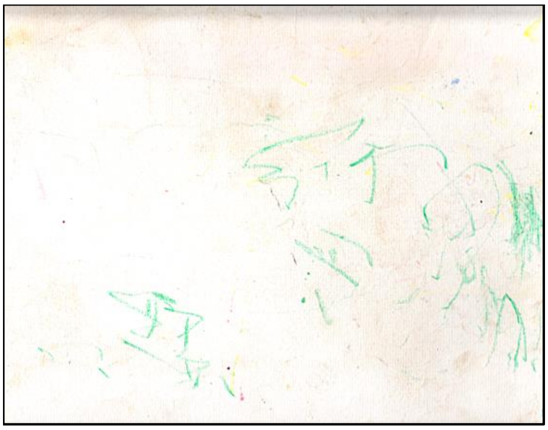

Jul. 28

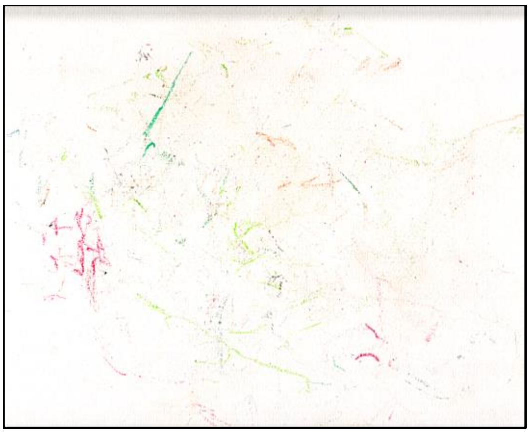

Mar. 5

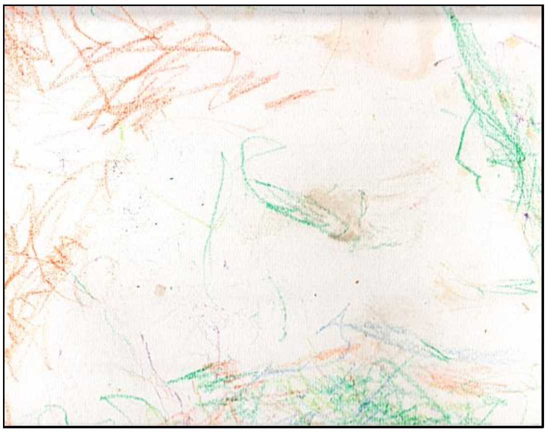

Oct. 5

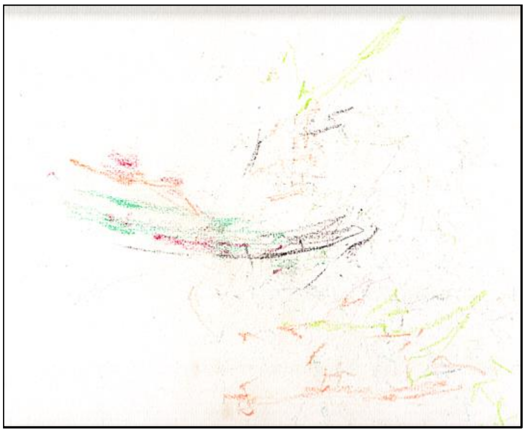

Mar. 3

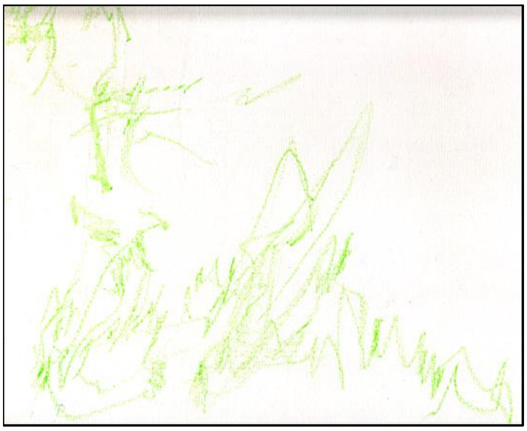

Feb. 10

References

Carne, C., Semple, S., and Lehmann, J. (2012). “The effects of climate change on orangutans: a time budget model,” in *Climate Models*, ed. L. Druyen (Rijeka: In-Tech Press), 313–336.

Hsu, S. H., Chuang, M. C., and Chang, C. C. (2000). A semantic differential study of designers’ and users’ product form perception. *Int. J. Ind. Ergon*. 25, 375–391. doi: 10.1016/S0169-8141(99)00026-8

Japan Meteorological Agency, (2007). *JMP Databases. Japan Meteorological Agency*. Available at: http://www.jma.go.jp/jma/indexe.html (accessed September 25, 2014).

Park, B. J., Furuya, K., Kasetani, T., Takayama, N., Kagawa, T., and Miyazaki, Y. (2011). Relationship between psychological responses and physical environments in forest settings. *Landsc. Urban Plan*. 102, 24–32. doi: 10.1016/j.landurbplan.2011.03.005

Suzuki, M., Gyoba, J., and Sakuta, Y. (2005). Multichannel NIRS analysis of brain activity during semantic differential rating of drawing stimuli containing different affective polarities. *Neurosci. Lett*. 375, 53–58. doi: 10.1016/j.neulet.2004.10.065
